# Biogeography and Genetic Structure in Populations of a Widespread Lichen (*Parmelina tiliacea*, *Parmeliaceae*, *Ascomycota*)

**DOI:** 10.1371/journal.pone.0126981

**Published:** 2015-05-11

**Authors:** Jano Núñez-Zapata, Paloma Cubas, David L. Hawksworth, Ana Crespo

**Affiliations:** Departamento de Biología Vegetal II, Facultad de Farmacia, Universidad Complutense de Madrid, Madrid, Spain; Ruhr-University Bochum, GERMANY

## Abstract

The genetic diversity and population structure of the foliose lichenized fungus *Parmelina tiliacea* has been analyzed through its geographical range, including samples from Macaronesia (Canary Islands), the Mediterranean, and Eurosiberia. DNA sequences from the nuclear ribosomal internal transcribed spacer, the mitochondrial large subunit ribosomal RNA gene, and the translation elongation factor 1-α were used as molecular markers. The haplotypes of the three markers and the molecular variance analyses of multilocus haplotypes showed the highest diversity in the Canary Islands, while restricted haplotypes occurred at high frequencies in Mediterranean coastal samples. The multilocus haplotypes formed three unevenly distributed clusters (clusters 1-3). In the Canary Islands all the haplotypes were present in a similar proportion, while the coastal Mediterranean sites had almost exclusively haplotypes of cluster 3; cluster 2 predominated in inland Mediterranean sites; and cluster 1 was more abundant in central and northern Europe (Eurosiberian area). The distribution of clusters is partially explained by climatic factors, and its interaction with local spatial structure, but much of the variation remains unexplained. The high frequency of individuals in the Canary Islands with haplotypes shared with other areas suggests that could be a refugium of genetic diversity, and the high frequency of individuals of the Mediterranean coastal sites with restricted haplotypes indicates that gene flow to contiguous areas may be restricted. This is significant for the selection of areas for conservation purposes, as those with most genetic variation may reflect historical factors and biological properties of the species.

## Introduction

Many lichenized fungi have long been considered to have exceptionally wide areas of distribution. However, detailed molecular studies are increasingly showing that, in some cases, different lineages are hidden under one species name, allowing recognition of more than one species [1]. Further, in other cases, a single lineage distributed over a wide area may display major genetic variation and geographical differentiation in the populations [2–3]. Large-scale differences in population structure may be explained by different post-glacial histories of coastal and inland populations [4], persistence in glacial refugia and subsequent genetic drift [5] or southern refugia during the last glaciations and subsequent recolonization into central and northern Europe [6]. In other cases, the genetic structure of the populations has been related to the interaction of climatic and geographical factors, with locally adapted algal partners and isolation by distance of the fungal ones [2, 7–8]. Additionally, for lichens growing mostly on trees, changes in the vegetation may affect the populations, for example by fragmentation of the distributional area of the host tree, either naturally or by human action [9–12].

*Parmelina tiliacea* is a foliose lichen occurring throughout Europe, North Africa, and the Canary Islands, and extending eastward to Asia. We decided to investigate the genetic structure within this species to ascertain if it showed any genetic variation. We hypothesized that this could be the case as the lichen occurs from sea level to 1500 m (and even to 3200 m in India), and under different ecological conditions. It is found on nutrient-rich bark of broad-leaved deciduous or coniferous trees, acid or siliceous rocks, as well as roofing tiles and memorials in well-lit situations. In Europe the species is particularly common around the Mediterranean, especially in central Iberia, extending through central into northern Europe, becoming rare in northern and western Scotland and with scattered localities in Ireland and southern Scandinavia. This species has asexual propagules (isidia, with both the algal and fungal partner), and also reproduces sexually by ascospores, although the spore-producing apothecia are often scarce or absent.

We used samples from a wide geographic range and selected three molecular markers of the fungal partner to build haplotype networks, analyze the population structure through clustering models and molecular variance analyses, and explore the geographic and climatic distribution of the global genetic variation of this widespread species. We did not use material previously recognized as a separate cryptic species, *P*. *cryptotiliacea* [13], in this study.

The study aimed to: (1) establish whether *P*. *tiliacea* consists of a single widely distributed lineage or shows any population subdivision; (2) determine if any genetic differentiation found corresponds with different geographical areas or other discontinuities that might be barriers to dispersal; and (3) ascertain if the distribution of any group of panmictic individuals correlates with, for example, macroclimatic variables or spatial trends. The analyses detected differences in haplotype composition between the regions, and allowed recognition of three clusters of populations in *P*. *tiliacea*. These clusters were partly explained by geography, local spatial structure, and climate variables. We also discuss the possible influence of past climate and vegetation.

## Material and Methods

### Taxon sampling

We studied 364 samples of *Parmelina tiliacea* from 62 localities representing different altitudes and habitats. No specific permissions were required for collecting at these locations because this species is very common and neither endangered nor protected. Sampling sites were from three broad geographical areas: Macaronesian (MA), Mediterranean *s*.*l*. (sensu [14]) and Eurosiberian (EU). EU included the Alpine, Atlantic, Boreal and Continental biogeographical regions as defined by [15]. Sites from the most extensively sampled area, the Mediterranean, were separated into two subgroups, one comprising inland (MI: Mediterranean-Inland) and the other coastal localities (MC: Mediterranean-Coastal). Details of localities, voucher specimens, and GenBank accession numbers are given in S1 Table. Nineteen bioclimatic variables of the 62 sampling sites were extracted from the 30 arc seconds resolution (~1 km) layers in the WorldClim repository (http://www.worldclim.org/) with Diva-GIS 7.5 [16].

### Molecular methods

DNA extraction and amplification of the nuclear ribosomal internal transcribed spacer (ITS) and partial large subunit mitochondrial ribosomal rRNA gene (mtLSU) followed the procedures described in [13] with some modifications: primers ITS1F [17] and ITS4A [18] were also used for amplification of ITS; and for the mtLSU region a second protocol was implemented, decreasing the annealing temperature by 1°C per 6 cycles from 63°C to 58°C, followed by 34 cycles of annealing at 40°C for 30 sec. A partial sequence of the translation elongation factor 1-α (*EF1-α*) was amplified using primers EFA-F and EFA-R [19], with similar parameters as for ITS but with an annealing temperature of 52 °C. PCR products were cleaned using ExoSAP-IT (USB, Cleveland, Ohio, USA), following manufacturers’ instructions. Sequencing was performed using the ABI Prism Dye Terminator Cycle Sequencing Ready Reaction Kit (Applied Biosystems, Life Technologies) with the PCR primers. Sequencing products were electrophoresed on a 3730 DNA analyzer (Applied Biosystems, Life Technologies) at the Unidad de Genómica (Parque Científico de Madrid). The sequence fragments obtained were assembled and manually adjusted in BioEdit 7.0.9.0 [20]. Three separate matrices (one for each locus) were built. Sequences were aligned using the ClustalW Multiple alignment program [21], and manually inspected.

### Individual loci, haplotype networks and multilocus analyses

Measures of genetic diversity were calculated for each sampling site and for the four geographic areas: the number of polymorphic sites (s), number of haplotypes (h), haplotype diversity (Hd) and nucleotide diversity (π) were estimated with DnaSP v5 [22] for each gene, and Tajima’s D tests were performed with the same program to test for selective neutrality [23]. To show the relatedness of haplotypes, we constructed median-joining haplotype networks for the three DNA loci using Hamming distance with the *haplonet* function of the *pegas* package [24], available through the Comprehensive R Archive Network (CRAN) [25]. The distribution of haplotypes with respect to geographical areas was displayed graphically on a network of each locus.

To assess whether the ITS, mtLSU and *EF1-α* loci could be combined in a joint analysis, we used the congruence among distance matrices (CADM) test [26] based on Kimura 2-parameter distance matrices [27] with function *CADM*.*global* implemented in the R library *ape* [28] which calculates the Kendall's coefficient of concordance *W* [29–30]. The null hypothesis of complete incongruence among DNA distance matrices was tested with 999 permutations in R. Tests to detect recombination events (RDP, GENECONV, CHIMAERA, MAXCHI) were performed for each locus with the options implemented in RDP3 [31], and the Perl script IMgc [32].

A matrix with the multilocus haplotypes (MLHs) of the three combined loci was built for each sampling site and AMOVA analyses were run with Arlequin 3.5.1.3 [33] to estimate the apportionment of genetic variation within and between sites and areas. The analyses run included: (a) all samples; and (b) non-redundant MLHs within sites to correct for any clonal signal. We investigated isolation by distance (IBD) using a Mantel test between MLHs genetic distances (D_R_, Rogers distance) and geographic distances of the sampling sites with the *mantel*.*randtest* function of the *adegenet* 1.4–1 package [34–35]. Correlations between these distances, however, can be due to IBD, which results in either continuous clines of genetic differentiation, or to the existence of distant and differentiated populations (distant patches). We plot local densities of distances to disentangle both processes, which were measured using a 2-dimensional kernel density estimation (function *kde2d)* and the results displayed with a customized color palette using *image* in MASS package [36].

### Population structure analyses

Clustering analyses were performed with Structure v2.3 [37] using the matrix of MLHs for inference of the number of clusters (k) and assignation of individual thalli to genepools. We used MLHs instead of sequences to avoid data being dominated by one or a few non- or low-recombining regions [38]. The analyses were run with an admixture model to allow individuals to have ancestry from multiple populations, a uniform prior of individual admixture from all genepools, and independent panmictic genepool allele frequencies [39]. We performed three replicate runs for each value of k (between 1 and 10), with 10^5^ iterations after a burn-in period of 10^4^ iterations. Analyses were run with a data set that included all 364 individuals, and with a collapsed data set excluding redundant haplotypes within sites. The best k value was selected based on the rate of change in the log likelihood of the data between successive k values runs (ΔK) using the methods in [40], through the Structure Harvester portal (http://taylor0.biology.ucla.edu/structureHarvester/). We used Distruct V1.1 [41] to graphically represent the individual assignment output of Structure. Individuals with over 85% probability of ancestry in a given gene pool were regarded as ‘assigned’ to that gene pool, whereas all other individuals were classified as ‘unassigned’. We performed χ^2^ tests of heterogeneity to assess whether the observed frequencies of clusters differed significantly from the null expectation of equal frequency across areas.

### Genetic structure, climate and geography

We used redundancy analysis (RDA)-based variation partitioning [42–43] to determine the fraction of variation revealed in the clustered genetic structure of *P*. *tiliacea* explained by the macroclimate and geography. As descriptors of spatial relationships we used principal coordinates of neighbor matrices (PCNM) obtained by principal coordinate analysis of a truncated matrix of Euclidean (geographic) distances among the sampling sites using the PCNM R package [44]. The PCNMs with positive spatial correlation were retained for redundancy analysis as suggested by [45]. For the RDA analysis we used the proportion of assignment to the clusters of the sites as the response variables, and climatic variables and spatial PCNMs as explanatory variables. Prior to the RDA, the cluster data were Hellinger-transformed [46] using the *decostand* function in the R package *vegan* [47], and climatic and PCNM variables were selected with function *forward*.*sel* of the *packfor* R package, retaining only those that were significant following the double stopping criterion [48]. The significance of the fractions of interest was estimated with functions *varpart* and *rda* coupled with a permutation test (*anova*) implemented in *vegan* [47].

## Results

We generated 1086 new sequences (348 of ITS, 348 of mtLS, and 364 of *EF1-α*) and downloaded the rest (up to 364 sequences per locus) from GenBank. The alignments included 462 bp from ITS, 716 bp from mtLSU and 546 bp from *EF1-α*. No recombination events were detected in the single matrices with either RPD3 or Imgc programs. Genetic diversity indices obtained for each marker globally and by geographical area are detailed in Table 1. The three loci were polymorphic, yielding 25 ITS, 8 mtLSU, and 24 *EF1-α* haplotypes. ITS and *EF1-α* had similar values of haplotype diversity (Hd) in all samples and in the different areas, while mtLSU values were lower and heterogeneous in the different areas. The lowest values of nucleotide diversity for the three loci were in the MC area. Globally, the three loci appeared to be selectively neutral according to Tajima’s D values. The significant positive D value for the ITS from the MA samples and the mtLSU from the MI and EU samples might suggest sudden population contraction or balancing selection at these loci. The frequency of the haplotypes in the four regions, a summary of the number of restricted haplotypes (i.e. haplotypes found only in a single area) for each marker, and the percentage of samples with those haplotypes, are shown in Table 2 and S2 Table.

**Table 1:** Summary of genetic diversity statistics of ITS, mtLSU, and *EF1-α* sequences of *Parmelina tiliacea* in the four geographical areas considered.

		N	s	h	Hd	π	D
ITS	Overall	364	26	25	0.868	0.00682	-0.72235
	MA	68	9	7	0.801	0.00725	2.07518*
	MI	111	19	15	0.774	0.00518	-1.14458
	MC	82	14	12	0.705	0.00356	-1.29077
	EU	103	14	10	0.783	0.00681	0.25464
mtLSU	Overall	364	17	8	0.679	0.00657	1.93858
	MA	68	16	7	0.722	0.00690	1.41370
	MI	111	11	3	0.548	0.00624	2.94599**
	MC	82	1	2	0.178	0.00025	-0.13463
	EU	103	12	5	0.592	0.00730	3.34958**
*EF1-α*	Overall	364	44	24	0.838	0.01846	0.99951
	MA	68	35	10	0.765	0.01695	0.52846
	MI	111	36	14	0.739	0.01531	0.39475
	MC	82	25	11	0.748	0.00842	-0.57509
	EU	103	30	11	0.724	0.01683	1.23510

Macaronesian (MA), Mediterranean inland (MI) and coastal (MC), and Eurosiberian (EU) areas. N: number of individuals, s: number of polymorphic sites, h: number of haplotypes, Hd: Haplotype (gene) diversity, π: nucleotide diversity (average over loci), D: Tajima’s D (significance *: p < 0.05; **: p< 0.10).

**Table 2:** Summary of the distribution of haplotypes restricted to a single area (restricted haplotypes).

	Percentage of restricted haplotypes/number of haplotypes in the area.				Percentage of individuals with restricted haplotypes/individuals in the area			
	ITS	mtLSU	*EF1-α*	MLHs	ITS	mtLSU	*EF1-α*	MLHs
MA	14.3	42.8	30	67.6	1.5	5.9	4.4	5.9
MI	40	0	28.6	57.1	5.4	0	9.9	21.6
MC	50	0	36.6	55	48.8	0	13.1	51.2
EU	30	20.0	18.12	55	14.6	0.9	18.2	13.6

Macaronesian (MA), Mediterranean inland (MI) and coastal (MC), and Eurosiberian (EU) areas.

**Fig 1:**
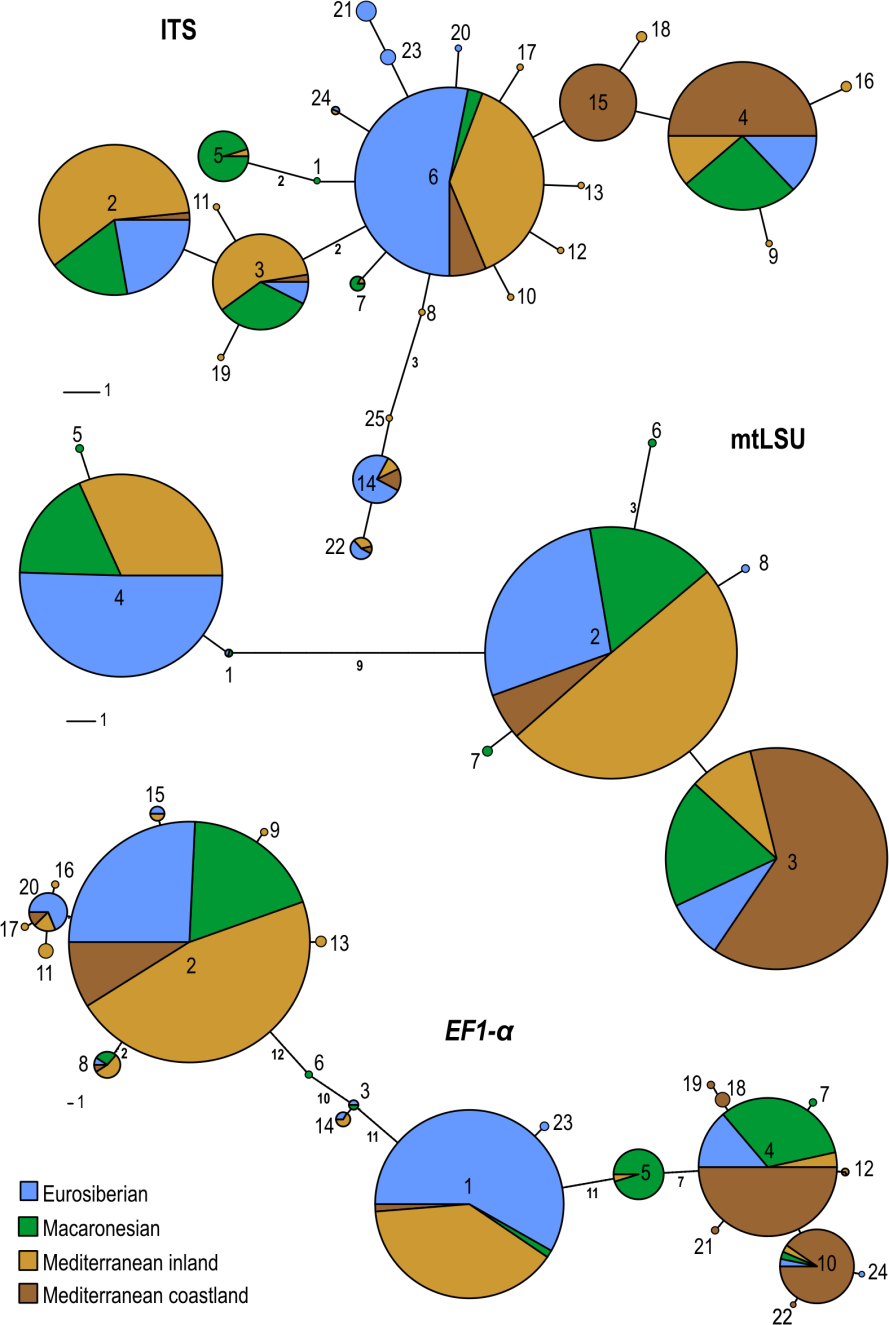
Median joining networks of three fungal markers. Pie charts numbered with haplotype codes as in S2 Table, proportional in size to the number of samples containing this haplotype, and colored according to the geographical area. Small numbers on lines indicate branch lengths.

In the ITS network (Fig 1) the four most frequent haplotypes (2, 3, 4, and 6) were connected by short branches, one to two changes in length. Many rare or restricted haplotypes were connected peripherally to the predominant ones. One ITS haplotype (15) was restricted to, and very frequent in, the MC area. In the mtLSU network, two very common haplotypes (2 and 3) were closely connected, while another frequent one (4) was separated by a long branch of nine changes. Haplotype 4 was not found in MC area, but was frequent in the others, and haplotype 3 predominated in MC. In the *EF1-α* network the dominant haplotypes were separated by very long branches with rarer haplotypes intermingled between them. Haplotype 10 was almost exclusive to MC. Overall, 4 ITS haplotypes, 2 mtLSU, and 5 *EF1-α* occurred in all the geographical areas, and 16 ITS, 4 mtLSU, and 13 *EF1-α* haplotypes were found in only a single area (S2 Table). In MC there was a high proportion of restricted ITS and *EF1-α* haplotypes, and no restricted mtLSU ones. In contrast, the MA area had a high proportion of mtLSU restricted haplotypes.

Kendall's coefficient of concordance (W) rejected the incongruence among the three matrices, globally (W = 0.5462059, *p* = 0.03) and by pairs, so we combined the loci into a multilocus haplotype (MLHs) matrix. Of the 78 MLHs found (S2 Table), only three (2, 3, and 4) were present in all the areas, and most were restricted to one. Two MLHs were very frequent and restricted: (1) MLH 5 was mostly restricted to MA; and (2) MLH 51 (19 samples) was confined to MC. The AMOVA of the MLHs matrix (Table 3) indicated little structure between the regions (Ф_RT_ = 0.17) when considering all four areas, but a very substantial structure (Ф_RT_ = 0.31) when MI sites were compared with MC ones. MC also had the highest values of pairwise differences (FSTs) against the MA, MI, and EU (0.20, 0.32 and 0.32, respectively; Table 4). Similar results were obtained when redundant haplotypes were excluded (0.12, 0.24 and 0.27, respectively).

**Table 3:** Analysis of molecular variance of multilocus haplotypes (MLHs) in *Parmelina tiliacea*.

	Source of variation	d.f.	Sum of squares	Variance components	Percentage of variation	Fixation indexes	*p*
Four regions, all samples	Between regions	3	69.754	0.211	16.84	0.168	<0.001
	Among sites within regions	58	181.093	0.444	35.39	0.426	<0.001
	Within sites	302	181.093	0.600	47.78	0.522	<0.001
	Total	363	432.827	1.255			
Four regions, redundant haplotypes excluded	Between regions	3	30.318	0.194	15.27	0.153	<0.001
	Among sites within regions	58	72.157	0.088	6.91	0.019	<0.001
	Within sites	124	122.433	0.987	77.82	0.222	<0.001
	Total	184	224.909	1.269			
Mediterranean regions (MI vs MC), all samples	Between regions	1	43.253	0.423	30.51	0.305	<0.001
	Among sites within areas	37	84.891	0.350	25.24	0.363	<0.001
	Within sites	154	94.510	0.614	44.25	0.558	<0.001
	Total	192	222.653	1.387			
Mediterranean regions (MI vs MC), redundant haplotypes excluded	Between regions	1	17.909	0.318	23.64	0.236	<0.001
	Among sites within areas	37	42.176	0.065	4.83	0.063	0.047
	Within sites	69	66.433	0.963	71.53	0.285	<0.001
	Total	107	126.519	1.346			

Genetic variation was partitioned among sites and regions, including all the samples or excluding redundant haplotypes within sites.

**Table 4:** Pairwise differences (FSTs) among areas.

	Area	MA	MI	MC
All samples	MI	0.10965		
	MC	0.20201	0.32323	
	EU	0.14049	0.05786	0.31819
Redundant haplotypes excluded	MI	0.04685	-	
	MC	0.12280	0.23890	
	EU	0.11596	0.06612	0.27459

Macaronesian (MA), Mediterranean inland (MI) and coastal (MC), and Eurosiberian (EU) areas.

**Fig 2:**
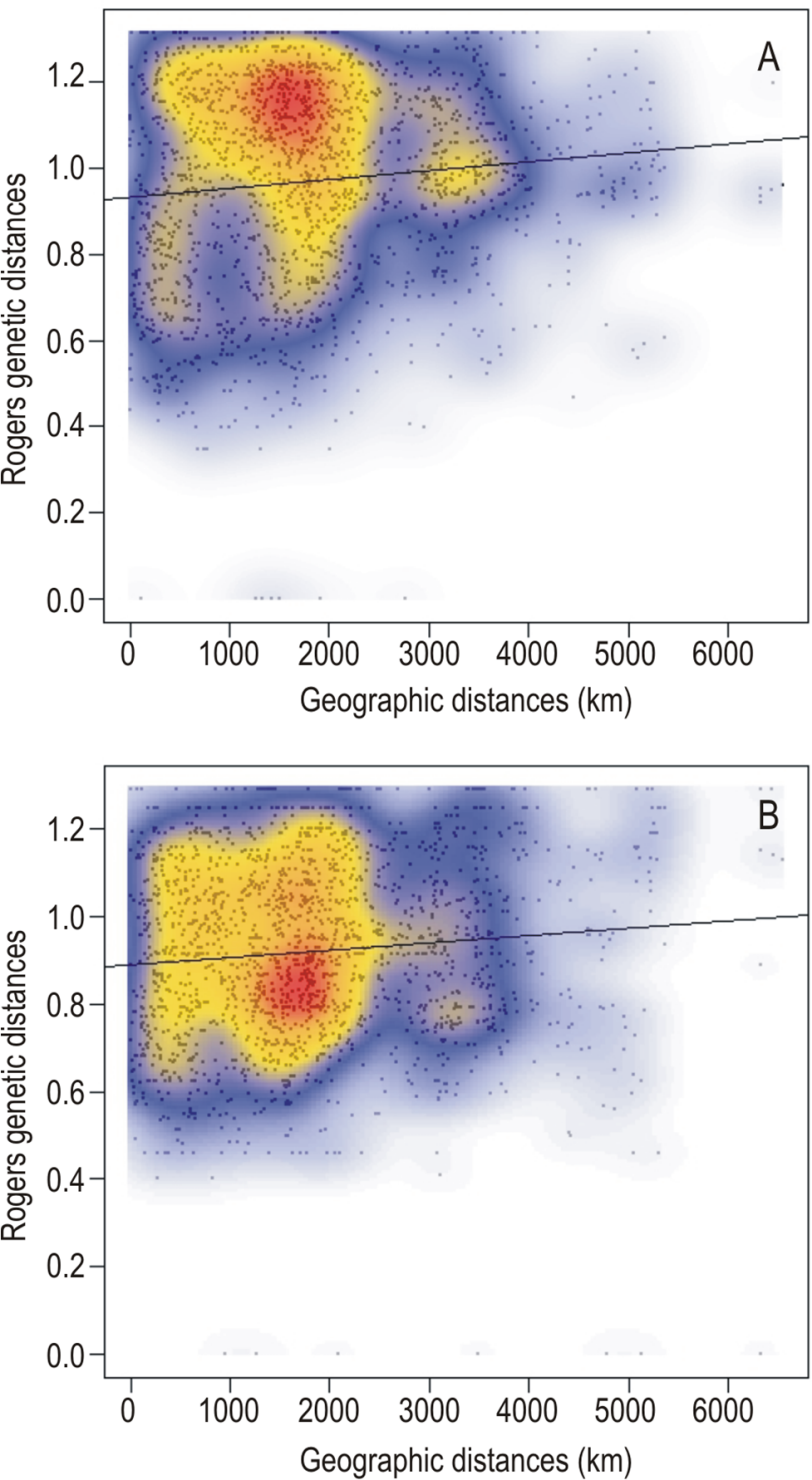
Isolation by distance plot. Scatterplots of local densities of Rogers genetic distances of MLHs against pairwise geographical distances of the sites. A) Including all samples per site, adjusted R^2^ = 0.01193, p < 0.001; and B) excluding redundant haplotypes per site, adjusted R^2^ = 0.00945, p < 0.001.

A weak pattern indicating isolation by distance between populations was detected in Mantel random tests with all samples (r = 0.111, p = 0.003) and without redundant haplotypes (r = 0.09987512, p = 0.019). However, the scatterplots of local densities of distances (Fig 2) detected some degree of discontinuity. This indicated that the genetic differentiation of the populations showed an incipient patched pattern and did not form a continuous cline.

### Structure of populations

**Fig 3:**
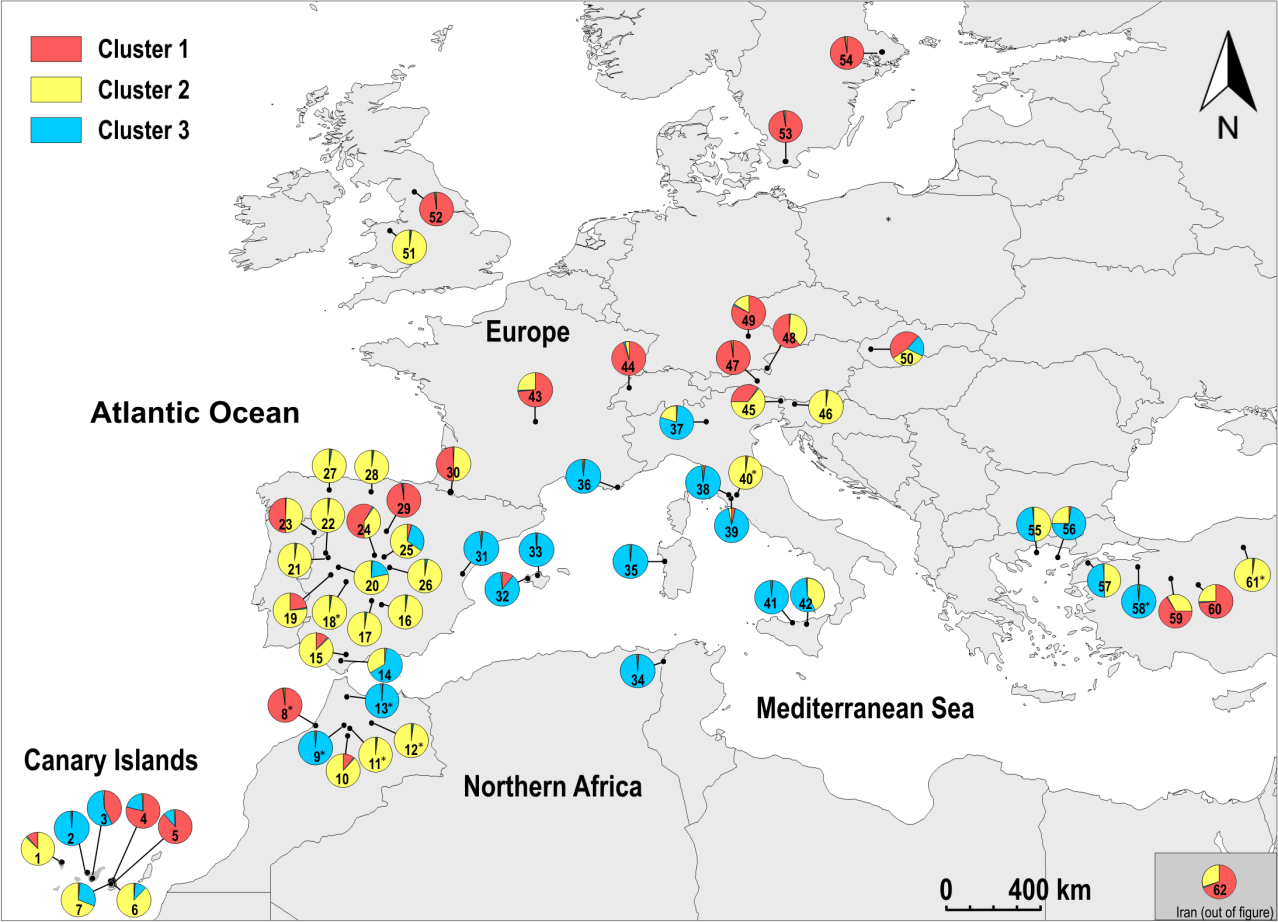
Distribution of the clusters at each site. Proportion of membership of each site in the three clusters inferred by Structure. Collection sites numbered as in S1 Table. Localities where only one individual was studied are marked with an asterisk.

**Fig 4:**
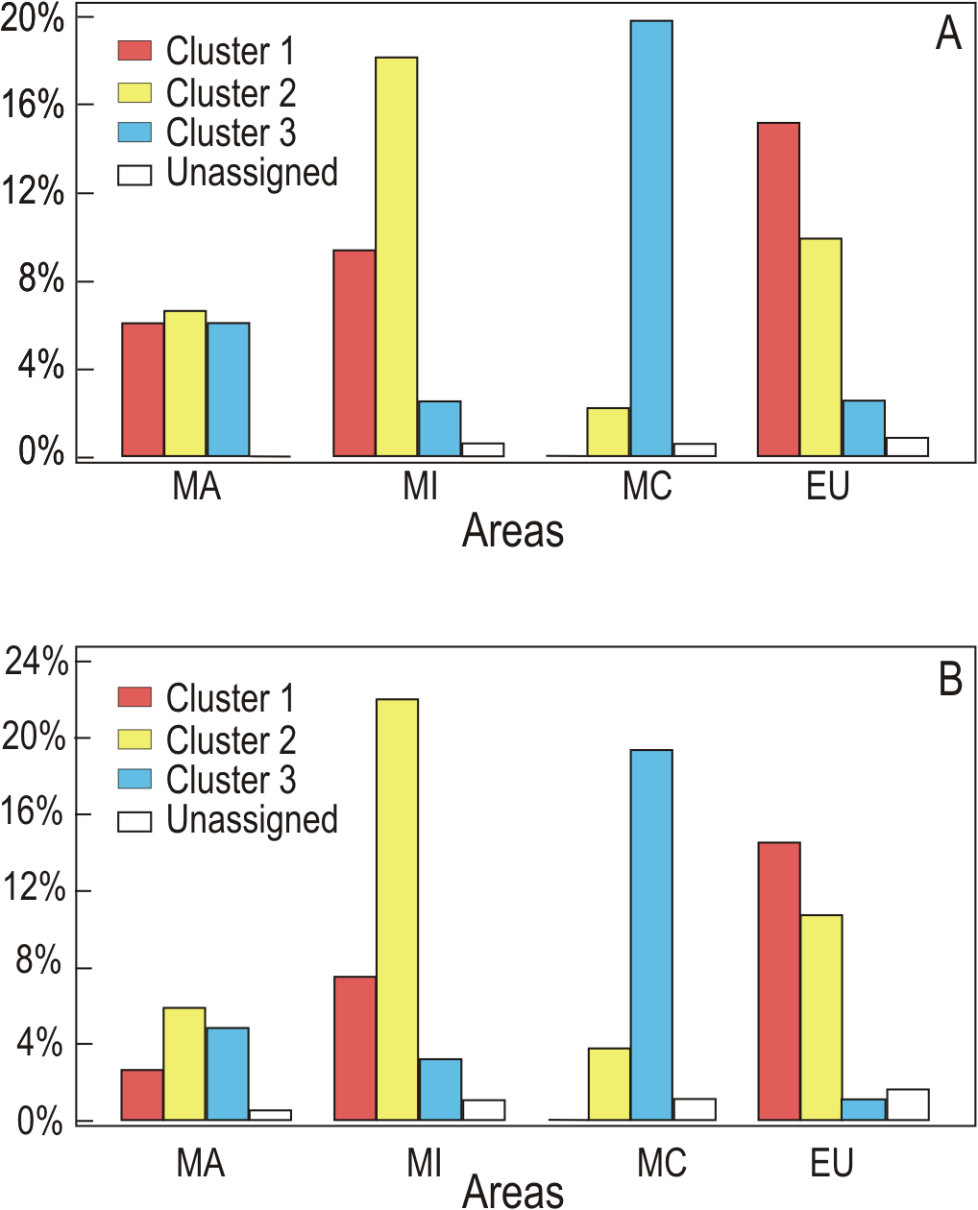
Percentage of the 357 individuals assigned to the different clusters and areas Macaronesian (MA), Mediterranean inland (MI) and coastal (MC), and Eurosiberian (EU) areas. A) Including all samples per site, and B) excluding redundant haplotypes per site.

**Fig 5:**
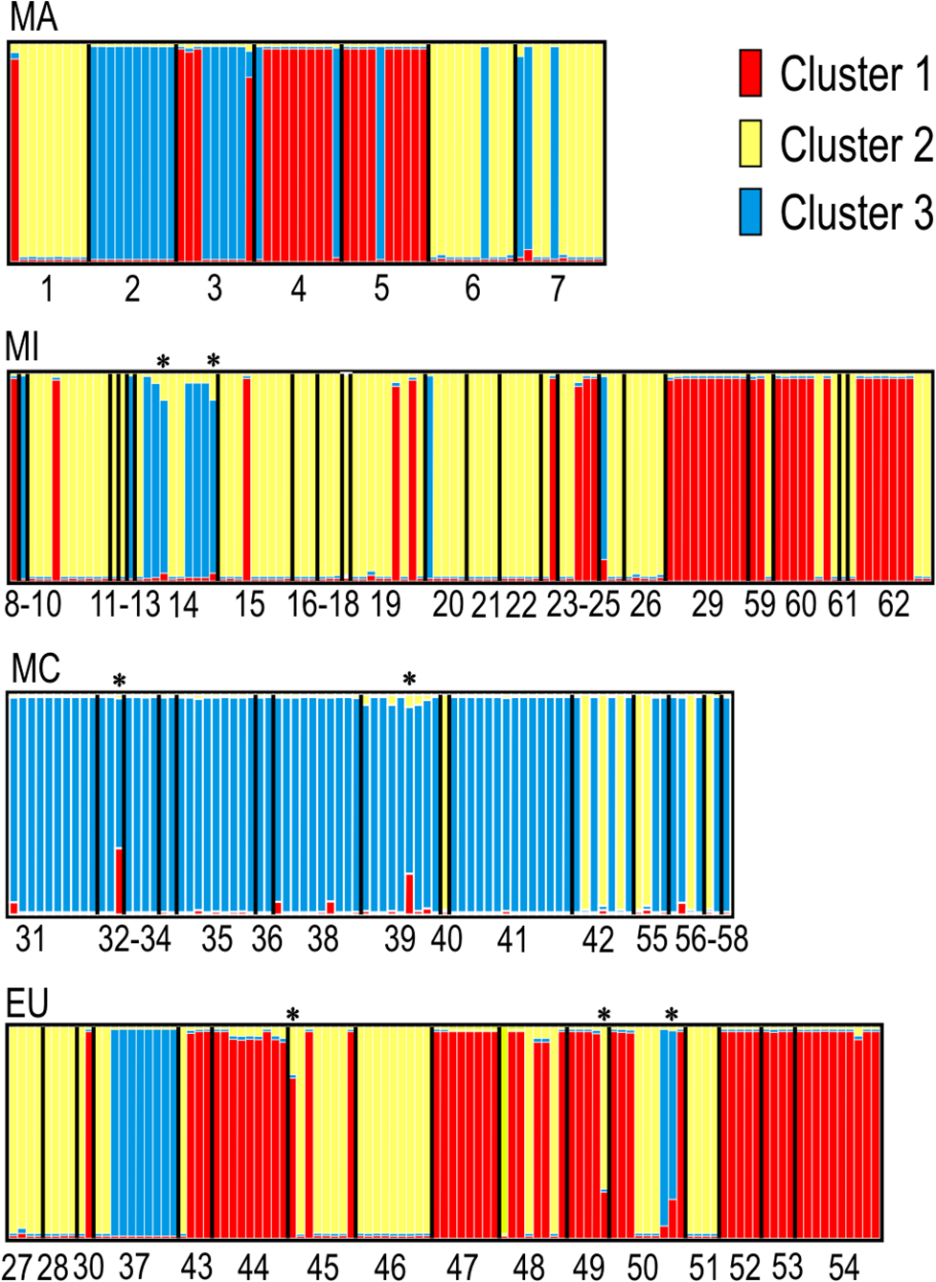
Inferred ancestry of individuals of *Parmelina tiliacea* from each site grouped by geographical areas. Collection sites numbered as in S1 Table. Each individual is represented by a single vertical line, broken into colored segments with lengths proportional to each of the three inferred clusters. Admixed individuals marked with an asterisk.

Structure analyses based on the distribution of the MLHs of the different sites showed that the best estimate for the number of clusters was k = 3. Similar groups were obtained when the analysis was run excluding redundant MLHs from each site. The proportion of the three clusters at the different sites is shown in Fig 3 and S3 Table. The distribution of the clusters across the geographical areas was heterogeneous (Table 5 and Fig 4). In the Canary Islands (MA), the three clusters were in similar proportions; at MI sites, especially in Iberia, the clusters were spatially intermingled, although cluster 2 had a higher frequency than the two other clusters; in EU, cluster 1 predominated but was intermingled with cluster 2; and most MC sites had cluster 3, except for some eastern ones where cluster 2 was also found. This last result must, however, be accepted with caution as we had few eastern Mediterranean samples. Of the 364 individuals, 357 were unequivocally assigned to a single cluster with a probability ≥ 85%, while seven showed admixed ancestry (Fig 5). Similar results were found when redundant haplotypes were excluded from the analysis (Table 5). In both analyses the hypothesis of independence between areas and clusters was rejected (p≤ 0.0001).

**Table 5:** Number of individuals assigned to each genetic cluster in each area.

	All individuals				Redundant haplotypes excluded			
	Cluster 1	Cluster 2	Cluster 3	Total	Cluster 1	Cluster 2	Cluster 3	Total
MA	22	24	22	68	5	11	9	25
MI	34	66	9	109	14	41	6	61
MC	0	8	72	80	0	7	36	43
EU	55	36	9	100	27	20	2	49
Total	111	134	112	357	46	79	53	178

Macaronesian (MA), Mediterranean inland (MI) and coastal (MC), and Eurosiberian (EU) areas.

### Relative contribution of climate and geography to the genetic variation

**Fig 6:**
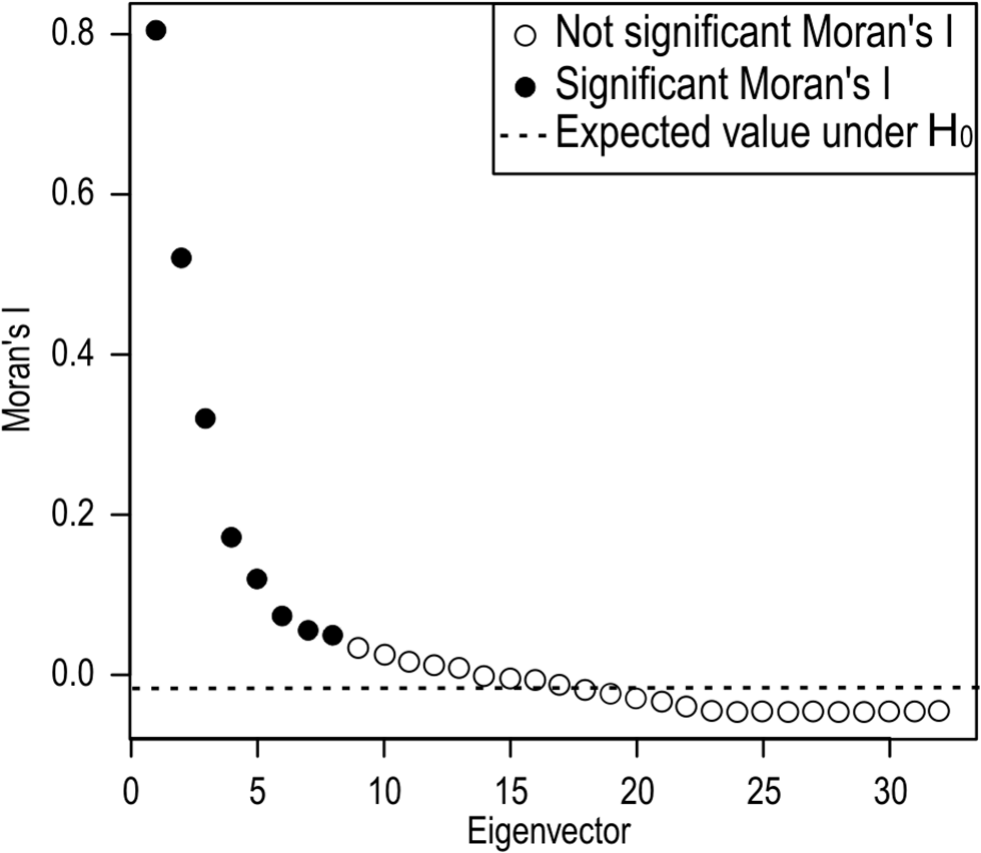
Moran’s I index of the cluster structure of *Parmelina tiliacea*.

A preliminary redundancy analysis (RDA) of the cluster data showed that the X and Y geographical coordinates were not significant, whereas three PCNM variables (accounting for fine scale patterns) had a positive Moran’s I index of spatial autocorrelation at low distances (Fig 6). Five bioclimatic variables were also found to be significant and selected for the analysis: BIO3 (isothermality), BIO6 (minimum temperature of the coldest month), BIO9 (mean temperature of the driest quarter), BIO13 (precipitation of the wettest month), and BIO16 (precipitation of the wettest quarter). The relative contribution of climate and fine spatial distribution to the variance of the clusters is shown in Table 6. Climate accounted for 19% of the variation of the cluster structure, and the interaction of fine scale patterns and climate for another 17%. A similar analysis using the genetic cluster structure without redundant haplotypes at the different sites (Table 7) was undertaken to exclude the clonal signal. In this analysis four climatic variables (BIO3, BIO6, BIO9 and BIO13) and five PCNM were used. The proportion of variation related to climate was slightly lower (15%) and the interaction of climate with geography slightly higher (19%). Both analyses indicated that climate and the interaction between climate and local spatial patterns explained about 35% of the genetic variation.

**Table 6:** Summary of variance partitioning analysis of cluster structure including all samples.

		d.f.	R^2^	Adj R^2^	Pr(>F)
Interactions	Climate+Interaction	5	0.41679	0.36472	—
	Geographyl+Interaction	3	0.23370	0.19406	—
	Climate+Geography+Interaction	8	0.46652	0.38600	
Individual fractions	Climate	5	—	0.19194	0.005^(**)^
	Interaction	0	—	0.17278	—
	Geography	3	—	0.02128	0.115
	Residuals			0.61400	—

Two explanatory matrices were used with five climatic variables (BIO3, BIO6, BIO9, BIO13, and BIO16) and three spatial (PCNM) variables. Total variation (SS): 24.327. Variance: 0.3988. Significance codes: **≤ 0.01,^.^ ≤0.1

**Table 7:** Summary of variance partitioning analysis of cluster structure excluding redundant haplotypes.

		d.f.	R^2^	Adj R^2^	Pr(>F)
Interactions	Climate+Interaction	4	0.38377	0.34053	—
	Geographyl+Interaction	5	0.29843	0.23579	—
	Climate+Geography+Interaction	9	0.47427	0.38327	
Individual fractions	Climate	4	—	0.14748	0.005^(**)^
	Interaction	0	—	0.19305	—
	Geography	5	—	0.04275	0.077 ^(.)^
	Residuals			0.61673	

Two explanatory matrices were used with four climatic variables (BIO3, BIO6, BIO9, BIO13) and five spatial variables (PCNM). Total variation (SS): 22.878. Variance: 0.37505. Significance codes: **≤ 0.01,^.^ ≤0.1

## Discussion

The three molecular markers of the fungal partner we used (ITS, mtLSU, and *EF1-α*) demonstrated genetic variability within *Parmelina tiliacea*. The ITS region is frequently used for phylogenetic studies in fungi and it has been adopted as a universal DNA barcode marker for species [49], although it does not discriminate between all taxa [50–51]. In our case, the ITS marker detected intraspecific genetic differences even at the same locality. Some ITS haplotypes were frequently found in well separated areas. However, in MC up to 50% of the haplotypes were restricted to this zone, and one of those was very frequent. Conversely, in MA most of the haplotypes were shared with other regions. The mtLSU showed lower variability than the ITS, as has been observed in some other lichen-forming fungi [2, 5]. In *P*. *tiliacea* this marker detected only three main haplotypes, one absent and another predominant in the MC area, as well as many restricted haplotypes at low frequencies in MA. The *EF1-α* marker, which has not often been used in studies of the population genetics of lichenized fungi [52–53], showed a high level of variability. Here again, the MC area showed a higher proportion of restricted haplotypes. The three networks had haplotypes present in all regions, and in addition others which were either restricted to, or unevenly distributed amongst, the regions. Further, MC had a high proportion of restricted ITS and *EF1-α* haplotypes, but none of mtLSU. In contrast, MA had a high proportion of mtLSU restricted haplotypes.

The combined MLHs also indicated that the Canary Islands (MA) had the highest number of restricted and rare MLHs, but most of the individuals had haplotypes widespread in other areas. On the contrary, the high number of exclusive MLHs found in the coastal Mediterranean sites (MC) were present in many individuals. Although the relative contribution of asexual and sexual processes in *P*. *tiliacea* remains unknown, the three markers detected genetic variability between and within populations. This would be expected in an organism with at least occasional sexual reproduction. The asexual propagules (isidia) can be expected to be dispersed within short distances from the parents as in other lichens [54] forming clonal populations. This is indicated by the number of redundant haplotypes found at some sites.

*Parmelina tiliacea* were subdivided into three clusters with heterogeneous distributions between regions, the extreme cases being the Canary Islands (MA) and the coastal sites (MC) around the Mediterranean Basin (as opposed to more continental ones). In the Canary Islands (MA area), the three genetic clusters occurred in similar proportions, and there were also many restricted MLHs at very low frequencies, most individuals sharing MLHs with other areas. Our data indicate that these islands retain the greatest amount of diversity in this species and suggest that they may have served as a refugium for it. The Canary Islands, as other oceanic islands at low latitudes, are generally considered to have been buffered from the pronounced climate changes of the last 2 Ma [55], and to have served as a refugium and a source area for the postglacial recolonization of Europe by spore-producing plants (e.g. [56]).

In the coastal Mediterranean sites (MC), however, in all those around the Mediterranean Basin, cluster 3 was dominant and a very high proportion of the individuals had MLHs restricted to this area. This may indicate a geographic barrier for dispersal, as seems to occur with the Pyrenees and the Alps in the case of some plants and insects [57–58]. These differences could, however, also be a response to competition with other species, and the persistence (or colonization) of only the best adapted haplotypes to this region. It is conceivable that the eastern Mediterranean area could have been another refugium, as in plants [59–60], but more extensive sampling within that region would be needed to test that hypothesis.

The geographical genetic differentiation found cannot be explained by isolation by distance (IBD) alone, although a weak pattern of spatial autocorrelation at low distances was detected. Part of the variation of the cluster distribution can be explained by the macroclimatic variables alone, independently of any spatial structure. Another fraction reflects the interaction between climate and the local spatial structure generated by other natural processes, factors that are often mixed together. Both variables have been found to explain population structure in other lichens [2, 7]. Nevertheless, there is still a large percentage of the variation discovered in *P*. *tiliacea* that remains unexplained, and which could perhaps be related to intrinsic biological properties or ecological requirements of the species. Factors related to the historical biogeography of the species (particularly bottlenecks, founder effect, or glacial refugial areas) could also have been involved in the initiation of differentiation in this widespread species.

The macroclimatic variables we used are only rough approximations for the ecological requirements of the individuals, as they do not take into account either local microclimatic conditions that may be important for the establishment of lichens [61], or past climatic events that permitted the colonization of an area. Considering the estimated age (8.45 Ma) for the origin of the genus *Parmelina* [62], the diversification of *P*. *tiliacea* must be a relatively young process. Thus, the present structure of the continental populations (i.e. the non Macaronesian sites) could well have been influenced by Miocene and Pleistocene events, as suggested for some other organisms [57] and also lichens [63].

Our study highlights the special position of the *Parmelina tiliacea* populations in the Canary Islands and in the Mediterranean coastal sites in relation to the differentiation of lineages within species and the conservation of its genetic diversity. These two areas serve as significant reservoirs of its genetic diversity and so merit preservation. More discriminating markers and more detailed information on microclimatic variables are needed to establish the contribution of clonal *vs* sexual reproduction to the fine-scale structure of the populations. The contribution of the algae component to the establishment of a particular fungal genotype in a location has been demonstrated in other species [8], but this was beyond the scope of the present paper. It is, nevertheless, another factor which merits exploration in a future study.

## Supporting Information

S1 Table Locality information with herbarium codes and GenBank accessions of the samples used in the present study. (PDF)

S2 Table Frequency of the haplotyes of ITS, mtLSU, *EF1-α*, and MLHs. Macaronesian (MA), Mediterranean inland (MI) and coastal (MC), and Eurosiberian (EU) areas. Haplotypes exclusive of a single area in bold. PH: private haplotypes/number of haplotypes in the area. SPH: samples with private haplotype/number of samples in the area.
(PDF)

S3 Table Proportion of membership of each locality in clusters 1 to 3 inferred by Structure. The analyses were run with an admixture model and uncorrelated loci. Locality codes as in S1 Table and Fig 1; n = number of individuals.
(PDF)
