# Supplementary material for: Biogeography and Genetic Structure in Populations of a Widespread Lichen (Parmelina tiliacea, Parmeliaceae, Ascomycota)
Source: PLoS One. 2015 May 11;10(5):e0126981. doi: 10.1371/journal.pone.0126981 (PMC4427293; doi:10.1371/journal.pone.0126981)
Supplement: S1 Table — (PDF) [file pone.0126981.s001.pdf]

S1 Table. Locality information with herbarium codes and GenBank accessions of individuals used in the present study. Macaronesian (MA), Mediterranean inland (MI), Mediterranean coastal (MC) and Eurosiberian (EU) areas.

Code	Locality	No. indiv.	Longitude	Latitude	Altitude	Area	Herbarium	ITS GenBank Accession	mtLSU GenBank Accession	EFA GenBank Accession
	Parmelina tiliacea									
1	El Roque, La Palma, Canary Islands, Spain	9	-17.83	28.74	1993	MA	MAF-Lich 16469; 17231 - 17238	JX466130 - JX466138	JX466479 - JX466487	JX465753 - JX465761
2	Degollada del Teno Alto, Tenerife, Canary Islands, Spain	10	-16.86	28.34	829	MA	MAF-Lich 17239 - 17248	JX466139 - JX466148	JX466488 - JX466497	JX465762 - JX465771
3	La Escalona, Tenerife, Canary Islands, Spain	9	-16.67	28.12	982	MA	MAF-Lich 17249 - 17257	JX466149 - JX466157	JX466498 - JX466506	JX465772 - JX465780
4	Vega de San Mateo to Tenteniguada, Gran Canaria, Canary Islands, Spain	10	-15.53	28.00	976	MA	MAF-Lich 17277 - 17286	JX466177 -JX466186	JX466526 - JX466535	JX465801 - JX465810
5	Galdar, Gran Canaria, Canary Islands, Spain	10	-15.51	28.09	794	MA	MAF-Lich 16470; 17287 - 17295	JF756983; JX466187 - JX466195	JF757032; JX466536 - JX466544	JX465811 - JX465820
6	Degollada de la Becerra, Gran Canaria, Canary Islands, Spain	10	-15.59	27.99	1499	MA	MAF-Lich 17258 - 17267	JX466158 - JX466167	JX466507 - JX466516	JX465781 - JX465790
7	Valleseco, Gran Canaria, Canary Islands, Spain	10	-15.59	28.02	1305	MA	MAF-Lich 16482; 17268 - 17276	JF756984; JX466168 - JX466176	JF757033; JX466517 - JX466525	JX465791 - JX465800
8	Rabat, Rabat-Salé-Zemmour-Zaer, Morocco	1	-6.71	34.03	112	MI	MAF-Lich 17296	JX466196	JX466545	JX465821
9	Mulay Idriss, Meknès-Tafilalet, Morocco	1	-5.48	34.03	1045	MI	MAF-Lich 17306	JX466206	JX466555	JX465832
10	Ifrane, Meknès -Tafilalet, Morocco	10	-5.33	33.63	543	MI	MAF-Lich 16468; 17297 - 17305	JF756988; JX466197 - JX466205	JF757037; JX466546 - JX466554	JX465822 - JX465831
11	Ain Taoujdate, Meknès-Tafilalet, Morocco	1	-5.24	33.92	1375	MI	MAF-Lich 16486	JF756976	JF757025	JX465833
12	Taza, Taza-Al Hoceima-Taounate, Morocco	1	-4.28	34.12	543	MI	MAF-Lich 17307	JX466207	JX466556	JX465834
13	Chauen, Tangier-Tetouan, Morocco	1	-5.36	35.14	580	MI	MAF-Lich 17308	JX466208	JX466557	JX465835
14	La Sauceda, Andalucía, Spain	10	-5.59	36.53	520	MI	MAF-Lich 17309 - 17318	JX466209 - JX466218	JX466558 - JX466567	JX465836 - JX465845
15	Grazalema, Andalucía, Spain	9	-5.38	36.76	945	MI	MAF-Lich 17319 - 17327	JX466219 - JX466227	JX466568 - JX466576	JX465846 - JX465854
16	Puertollano, Castilla-La Mancha, Spain	3	-3.84	38.67	666	MI	MAF-Lich 17328 - 17330	JX466228 - JX466230	JX466577 - JX466579	JX465855 - JX465857
17	San Quintín, Castilla-La Mancha, Spain	3	-4.28	38.82	660	MI	MAF-Lich 16457; 17331 - 17332	JF756986; JX466231 - JX466232	JF757035; JX466580 - JX466581	JX465858 - JX465860
18	Navalvillar, Extremadura, Spain	1	-5.38	39.55	673	MI	MAF-Lich17355	JX466256	JX466605	JX465884
19	Monfragüe, Extremadura, Spain	9	-6.06	39.83	323	MI	MAF-Lich 17333 - 17341	JX466233 - JX466241	JX466582 - JX466590	JX465861 - JX465869
20	Cuacos de Yuste, Extremadura, Spain	5	-5.73	40.10	650	MI	MAF-Lich 16465; 17342 - 17345	JX466242 - JX466246	JX466591 - JX466595	JX465870 - JX465874
21	Peña de Francia, Castilla y León, Spain	4	-6.16	40.51	1320	MI	MAF-Lich 17346 - 17349	JX466247 - JX466250	JX466596 - JX466599	JX465875 - JX465878

Code	Locality	No. indiv.	Longitude	Latitud.	Altitude	Area	Herbarium	ITS GenBank Accession	mtLSU GenBank Accession	EFA GenBank Accession
22	Morasverdes, Castilla y León, Spain	5	-6.28	40.64	794	MI	MAF-Lich 17350 - 17354	JX466251 -JX466255	JX466600 - JX466604	JX465879 - JX465883
23	Braganza, Alto Trás-os-Montes, Portugal	2	-6.77	41.44	640	MI	MAF-Lich 15243 - 15350	JF756981; JX466257	JF757030; JX466606	JX465885 - JX465886
24	El Escorial, Madrid, Spain	5	-4.15	40.57	1100	MI	MAF-Lich 16467; 17358 - 17361	JF756980; JX466260 - JX466263	JF757029; JX466609 - JX466612	JX465890 - JX465894
25	El Pardo, Madrid, Spain	3	-3.75	40.51	675	MI	MAF-Lich 16452; 17356 - 17357	JF756979; JX466258 - JX466259	JF757028; JX466607 - JX466608	JX465887 - JX46589
26	Montejo de la Sierra, Madrid, Spain	5	-3.53	41.06	1180	MI	MAF-Lich 17362 - 17366	JX466264 - JX466268	JX466613 - JX466617	JX465895 - JX465899
27	Saliencia, Principado de Asturias, Spain	4	-6.12	43.08	1325	EU	MAF-Lich 16462; 17379 - 17381	JX466283 - JX466286	JX466632 - JX466635	JX465915 - JX465918
28	La Lomba, Cantabria, Spain	4	-4.30	43.02	1132	EU	MAF-Lich 17375 - 17378	JX466279 - JX466282	JX466628 - JX466631	JX465910 - JX465913
29	Moral de Hornuez, Castilla y León, Spain	10	-3.62	41.49	1135	MI	MAF-Lich 16459 - 16460; 17367 - 17374	JX466269 - JX466278	JX466618 - JX466627	JX465900 - JX465909
30	Belagua, Navarra, Spain	2	-0.82	42.96	1596	EU	MAF-Lich 17382 - 17383	JX466287 - JX466288	JX466636 - JX466637	JX465920 - JX465921
31	Castellón, Valencia, Spain	10	-0.32	39.85	420	MC	MAF-Lich 17391 - 17400	JX466296 - JX466305	JX466645 - JX466654	JX465929 - JX465938
32	Banyalbufar, Mallorca, Balearic Islands, Spain	3	2.51	39.67	460	MC	MAF-Lich 17401 - 17403	JX466306 - JX466308	JX466655 - JX466657	JX465939 - JX465941
33	Ufanes, Mallorca, Balearic Islands, Spain	4	2.96	39.81	172	MC	MAF-Lich 16466; 17404 - 17406	JF756990; JX466309 - JX466311	JF757039; JX466658 - JX466660	JX465942 - JX465945
34	Al mawajin, Jendouba, Tunisia	2	8.31	36.49	698	MC	MAF-Lich 16458; 17407	JF756987; JX466312	JF757036; JX466661	JX465946 - JX465947
35	Badde Orca, Sardinia, Italy	9	8.48	40.35	522	MC	MAF-Lich 17408 - 17416	JX466313 - JX466321	JX466662 - JX466670	JX465948 - JX465956
36	Saint-Tropez, Provence-Alpes-Côte d'Azur, France	2	6.43	43.20	35	MC	MAF-Lich 16464; 17417	JF756982; JX466322	JF757031; JX466671	JX465957 - JX465958
37	Lodrino, Lombardia, Italy	10	10.28	45.72	710	EU	MAF-Lich 16483; 17455 - 17463	JF756985; JX466360 - JX466368	JF757034; JX466709 - JX466717	JX465996 - JX466005
38	Poggioferro, Toscana, Italy	10	11.35	42.69	462	MC	MAF-Lich 17440 - 17449	JX466345 - JX466354	JX466694 - JX466703	JX465981 - JX465990
39	Ansedonia, Toscana, Italy	9	11.27	42.41	50	MC	MAF-Lich 17431 - 17439	JX466336 - JX466344	JX466685 - JX466693	JX465972 - JX465980
40	Monte Amiatta, Toscana, Italy	1	11.60	42.90	1308	MC	MAF-Lich 17450	JX466355	JX466704	JX465991
41	Santuario de Gibilmanna, Sicily, Italy	14	14.02	37.99	792	MC	MAF-Lich 19200 - 19213	KP659519 - KP659532	KP659563 - P659576	KP659475 - KP659488
42	Messina, a lo largo de la carretera SS289, km 27, Sicily, Italy	7	14.63	37.94	1183	MC	MAF-Lich 19193 - 19199	KP659533 - KP659539	KP659577 - KP659583	KP659489 - KP659495
43	Château de Cordès, Auvergne, France	4	2.85	45.70	899	EU	MAF-Lich 17418 - 17421	JX466323 - JX466326	JX466672 - JX466675	JX465959 - JX465962
44	Neuchâtel, Neuchâtel, Switzerland	9	6.91	47.00	665	EU	MAF-Lich 17422 - 17430	JX466327 -JX466335	JX466676 - JX466684	JX465963 - JX465971
45	Camporosso in Valcanale, Friuli-Venecia Julia, Italy	8	13.53	46.51	833	EU	MAF-Lich 17474 - 17481	JX466379 - JX466386	JX466728 - JX466735	JX466016 - JX466023

Code	Locality	No. indiv.	Longitude	Latitud.	Altitude	Area	Herbarium	ITS GenBank Accession	mtLSU GenBank Accession	EFA GenBank Accession
46	Bled, Upper Carniola, Slovenia	9	14.11	46.37	475	EU	MAF-Lich 16619; 17484 - 17491	JF756992; JX466389 - JX466396	JF757041; JX466738 - JX466745	JX466026 - JX466034
47	Mittersill, Salzburg, Austria	8	12.48	47.27	812	EU	MAF-Lich 16618; 17492 - 17498	JF756991; JX466397 - JX466403	JF757040; JX466746 - JX466752	JX466035 - JX466042
48	Bad Reichenhall, Upper Bavaria, Germany	8	12.93	47.76	463	EU	MAF-Lich 17499 - 17506	JX466404 - JX466411	JX466753 - JX466760	JX466043 - JX466050
49	Regensbrug, Bavaria, Germany	5	12.09	49.00	705	EU	MAF-Lich 16485; 17509 - 17512	JF756989; JX466414 - JX466417	JF757038; JX466763 - JX466766	JX466053 - JX466057
50	Smolenice, Trnava, Slovakia	9	17.43	48.50	250	EU	MAF-Lich 19214 - 19222	KP659540- KP659548	KP659584- KP659592	KP659496- KP659504
51	Rhydlydan, Wales, United Kingdom	4	-3.47	53.04	320	EU	MAF17528 - 17531	JX466443 - JX466446	JX466792 - JX466795	JX466085 - JX466088
52	Sandford, Cumbria, North West England, United Kingdom	5	-2.41	54.54	137	EU	MAF-Lich 17535 - 17539	JX466450 - JX466454	JX466799 - JX466803	JX466092 - JX466096
53	Sjobo, Skåne, Sweden	4	13.7	55.64	50	EU	MAF-Lich 19223 - 19226	KP659549 - KP659552	KP659593 - KP659597	KP659505 - KP659508
54	Uppsala, Uppland, Sweden	10	17.63	59.85	25	EU	MAF-Lich 17548 - 17557	JX466463 - JX466472	JX466812 - JX466821	JX466105 - JX466114
55	Maries, Thasos Island, Greece	4	24.62	40.68	537	MC	B600183598; B600183667;B600183986; B600186705	JX466418 - JX466421	JX466767 - JX466770	JX466058 - JX466061
56	Daphnes, Samothraki Island, Greece	4	25.55	40.51	50	MC	B600184168; B600185102; B600185118; B600186947	JX466423 - JX466426	JX466772 - JX466775	JX466063 - JX466066
57	Dumanli, Canakale, Turkey	2	26.88	40.28	670	MC	MAF-Lich 17513 - 17514	JX466428 - JX466429	JX466777 - JX466778	JX466068 - JX466069
58	Bursa, Uludaj, Turkey	1	29.03	40.13	1030	MC	MAF-Lich 16456	JF756977	JF757026	JX466072
59	Asagi Caglan, Eskisehir, Turkey	3	30.48	39.68	960	MI	MAF-Lich 17516 - 17518	JX466431 - JX466433	JX466780 - JX466782	JX466073 - JX466075
60	Sivrihisar Mountains, Eskisehir, Turkey	8	31.67	39.42	1040	MI	MAF-Lich 17519 - 17526	JX466434 - JX466441	JX466783 - JX466790	JX466076 - JX466083
61	Inköyü, Cankiri, Turkey	1	33.63	40.88	1200	MI	MAF-Lich 17527	JX466442	JX466791	JX466084
62	Aynaloo-Vaygan, Kaleybar county, Iran	10	46.82	38.88	2050	MI	MAF-Lich 19372 - 19381	KP659553 - KP659562	KP659597 - KP659606	KP659509 - KP659518
