# Supplementary material for: Biogeography and Genetic Structure in Populations of a Widespread Lichen (Parmelina tiliacea, Parmeliaceae, Ascomycota)
Source: PLoS One. 2015 May 11;10(5):e0126981. doi: 10.1371/journal.pone.0126981 (PMC4427293; doi:10.1371/journal.pone.0126981)
Supplement: S2 Table — Haplotypes exclusive of a single area in bold. PH: private haplotypes/number of haplotypes in the area. SPH: samples with private haplotype/number of samples in the area. (PDF) [file pone.0126981.s002.pdf]

S2 Table. Frequency of the haplotypes of ITS, mtLSU, EF1-α, and MLH. Area codes: Macaronesian (MA), Mediterranean inland (MI) and coastal (MC), and Eurosiberian (EU) areas. Haplotypes exclusive of a single area in bold. RH: restricted haplotypes/number of haplotypes in the area. RUH: samples with restricted haplotypes/number of samples in the area. Color codes: haplotypes present in all areas (gray), in three areas (red), and in two areas (blue).

Area / ITS	1	2	3	4	5	6	7	8	9	10	11	12	13	14	15	16	17	18	19	20	21	22	23	24	25	RH	SRH
MA	**1**	11	13	16	20	2	5	0	0	0	0	0	0	0	0	0	0	0	0	0	0	0	0	0	0	1/7	1/68
MI	0	37	23	7	1	30	1	**1**	**1**	**1**	**1**	**1**	**1**	2	0	0	0	0	0	0	0	3	0	1	0	6/15	6/111
MC	0	1	1	31	0	5	0	0	0	0	0	0	0	3	**32**	**4**	**1**	**1**	**1**	0	0	1	0	0	**1**	6/12	40/82
EU	0	14	3	8	0	42	0	0	0	0	0	0	0	15	0	0	0	0	0	**1**	**8**	5	**6**	1	0	3/10	15/103
Total samples	1	63	40	62	21	79	6	1	1	1	1	1	1	20	32	4	1	1	1	1	8	9	6	2	1	16/25	62/364

Area / mtLSU	1	2	3	4	5	6	7	8																		RH	SRH
MA	1	22	22	19	**1**	**1**	**2**	0	–	–	–	–	–	–	–	–	–	–	–	–	–	–	–	–	–	3/7	4/68
MI	0	66	11	34	0	0	0	0	–	–	–	–	–	–	–	–	–	–	–	–	–	–	–	–	–	0/3	0/111
MC	0	8	74	0	0	0	0	0	–	–	–	–	–	–	–	–	–	–	–	–	–	–	–	–	–	0/2	0/82
EU	1	37	10	54	0	0	0	**1**	–	–	–	–	–	–	–	–	–	–	–	–	–	–	–	–	–	1/5	1/103
Total	2	133	117	107	1	1	2	1	–	–	–	–	–	–	–	–	–	–	–	–	–	–	–	–	–	4/8	5/364

Area / EF1-α	1	2	3	4	5	6	7	8	9	10	11	12	13	14	15	16	17	18	19	20	21	22	23	24		RH	SRH
MA	1	19	2	19	20	**1**	**1**	3	**1**	1	0	0	0	0	0	0	0	0	0	0	0	0	0	0	–	3/10	3/68
MI	31	47	0	2	1	0	0	6	0	1	**6**	2	**3**	4	3	**1**	**1**	0	0	3	0	0	0	0	–	4/14	11/111
MC	1	9	0	29	0	0	0	1	0	28	0	1	0	0	0	0	0	**6**	**2**	2	**2**	**1**	0	0	–	4/11	11/82
EU	46	26	2	8	0	0	0	1	0	1	0	0	0	2	3	0	0	0	0	11	0	0	**2**	**1**	–	2/11	3/103
Total	79	101	4	58	21	1	1	11	1	31	6	3	3	6	6	1	1	6	2	16	2	1	2	1	–	13/24	28/364

Area / MLH	1	2	3	4	5	6	7	8	9	10	11	12	13	14	15	16	17	18	19	20	21	22	23	24	25	27	26
MA	**1**	8	**2**	16	19	**1**	**1**	**2**	**1**	1	**1**	9	1	**1**	**1**	1	1	**1**	0	0	0	0	0	0	0	0	0
MI	0	23	0	2	1	0	0	0	0	2	0	13	4	0	0	0	1	0	**1**	**4**	**1**	**4**	**1**	**1**	27	**3**	**2**
MC	0	1	0	20	0	0	0	0	0	0	0	1	0	0	0	2	0	0	0	0	0	0	0	0	0	0	0
EU	0	9	0	8	0	0	0	0	0	0	0	1	0	0	0	0	0	0	0	0	0	0	0	0	31	0	0
Total	1	41	2	46	20	1	1	2	1	3	1	24	5	1	1	3	2	1	1	4	1	4	1	1	58	3	2

	28	29	30	31	32	33	34	35	36	37	38	39	40	41	42	43	44	45	46	47	48	49	50	51	52	54	53
MA	0	0	0	0	0	0	0	0	0	0	0	0	0	0	0	0	0	0	0	0	0	0	0	0	0	0	0
MI	**1**	2	**1**	3	**1**	**1**	**1**	**2**	**1**	**1**	0	**1**	0	0	0	0	0	0	0	0	0	1	0	0	0	0	0
MC	0	0	0	0	0	0	0	0	0	0	0	0	0	**6**	**1**	**5**	**1**	**5**	**1**	**1**	**1**	2	2	**19**	**1**	0	0
EU	0	6	0	2	0	0	0	0	0	0	**1**	0	**1**	0	0	0	0	0	0	0	0	6	1	0	0	**3**	**1**
Total	1	8	1	5	1	1	1	2	1	1	1	1	1	6	1	5	1	5	1	1	1	9	3	19	1	3	1

	55	56	57	58	59	60	61	62	63	64	65	66	67	68	69	70	71	72	73	74	75	76	77	78		RH	SRH
MA	0	0	6	0	0	**1**	**5**	**3**	**1**	2	**5**	**2**	**2**	**1**	**1**	**1**	1	**2**	**3**	**1**	0	0	0	0	–	23/34	40/68
MI	0	0	0	0	0	0	0	0	0	0	0	0	0	0	0	0	0	0	0	0	0	0	0	0	–	17/28	24/111
MC	0	0	1	0	0	0	0	0	0	2	0	0	0	0	0	0	1	0	0	0	0	0	0	**1**	–	11/20	42/82
EU	**1**	**1**	1	**1**	**1**	0	0	0	0	0	0	0	0	0	0	0	0	0	0	0	**1**	**2**	**1**	0	–	11/20	14/103
Total	1	1	8	1	1	1	5	3	1	4	5	2	2	1	1	1	2	2	3	1	1	2	1	1	–	62/78	120/364
