# Supplementary material for: Biogeography and Genetic Structure in Populations of a Widespread Lichen (Parmelina tiliacea, Parmeliaceae, Ascomycota)
Source: PLoS One. 2015 May 11;10(5):e0126981. doi: 10.1371/journal.pone.0126981 (PMC4427293; doi:10.1371/journal.pone.0126981)
Supplement: S3 Table — The analyses were run with an admixture model and uncorrelated loci. Locality codes as in S1 Table and Fig 1; n = number of individuals. (PDF) [file pone.0126981.s003.pdf]

S3 Table. **Proportion of membership of each locality in clusters 1 to 3 inferred by Structure.** The analyses were run with an admixture model and uncorrelated loci. Locality codes as in S1 Table and Fig. 1; n = number of individuals.

	All the samples included					Redundant haplotypes excluded			
Locality	Cluster 1	Cluster 2	Cluster 3	n	Locality	Cluster 1	Cluster 2	Cluster 3	n
1	0.114	0.873	0.013	9	1	0.319	0.660	0.021	3
2	0.010	0.010	0.979	10	2	0.012	0.011	0.978	1
3	0.425	0.013	0.562	9	3	0.669	0.022	0.308	4
4	0.786	0.011	0.203	10	4	0.337	0.014	0.649	3
5	0.882	0.011	0.108	10	5	0.492	0.012	0.496	2
6	0.012	0.879	0.109	10	6	0.015	0.833	0.152	7
7	0.017	0.691	0.292	10	7	0.023	0.496	0.481	6
8	0.979	0.011	0.011	1	8	0.973	0.012	0.015	1
9	0.011	0.010	0.979	1	9	0.011	0.011	0.978	1
10	0.107	0.881	0.011	10	10	0.204	0.783	0.013	5
11	0.011	0.977	0.011	1	11	0.012	0.975	0.012	1
12	0.011	0.978	0.011	1	12	0.012	0.976	0.012	1
13	0.011	0.011	0.979	1	13	0.011	0.011	0.978	1
14	0.019	0.338	0.643	10	14	0.036	0.373	0.590	6
15	0.119	0.870	0.011	9	15	0.334	0.653	0.012	3
16	0.011	0.978	0.011	3	16	0.012	0.976	0.012	2
17	0.012	0.976	0.012	3	17	0.014	0.973	0.013	3
18	0.013	0.975	0.012	1	18	0.015	0.971	0.014	1
19	0.223	0.763	0.013	9	19	0.398	0.583	0.018	5
20	0.011	0.784	0.205	5	20	0.013	0.654	0.333	3
21	0.011	0.978	0.011	4	21	0.013	0.974	0.012	2
22	0.011	0.977	0.011	5	22	0.013	0.974	0.013	3
23	0.495	0.494	0.011	2	23	0.495	0.493	0.012	2
24	0.584	0.404	0.012	5	24	0.486	0.499	0.015	4
25	0.043	0.656	0.301	3	25	0.036	0.654	0.311	3
26	0.014	0.973	0.013	5	26	0.017	0.967	0.016	4
27	0.014	0.972	0.014	4	27	0.022	0.960	0.018	3
28	0.011	0.978	0.011	4	28	0.013	0.975	0.012	2
29	0.978	0.011	0.011	10	29	0.972	0.013	0.015	2
30	0.495	0.494	0.011	2	30	0.495	0.492	0.013	2
31	0.015	0.010	0.975	10	31	0.024	0.011	0.965	2
32	0.108	0.012	0.880	3	32	0.105	0.013	0.881	3
33	0.010	0.010	0.979	4	33	0.012	0.011	0.977	2
34	0.012	0.011	0.977	2	34	0.013	0.012	0.975	2
35	0.013	0.012	0.976	9	35	0.020	0.015	0.965	5
36	0.010	0.010	0.979	2	36	0.011	0.011	0.978	1
37	0.011	0.204	0.786	10	37	0.012	0.493	0.495	2
38	0.021	0.011	0.968	10	38	0.020	0.012	0.968	5
39	0.034	0.029	0.937	9	39	0.049	0.026	0.925	6
40	0.012	0.977	0.011	1	40	0.014	0.972	0.013	1
41	0.011	0.011	0.978	14	41	0.013	0.012	0.975	5
42	0.012	0.422	0.565	7	42	0.019	0.643	0.338	3
43	0.735	0.253	0.012	4	43	0.653	0.334	0.014	3
44	0.952	0.033	0.015	9	44	0.958	0.025	0.018	4
45	0.347	0.642	0.012	8	45	0.466	0.520	0.014	6
46	0.011	0.977	0.011	9	46	0.015	0.972	0.013	4
47	0.978	0.011	0.011	8	47	0.976	0.011	0.012	3
48	0.603	0.384	0.013	8	48	0.557	0.429	0.013	7
49	0.825	0.163	0.013	5	49	0.717	0.267	0.017	3
50	0.465	0.333	0.202	9	50	0.525	0.172	0.302	6
51	0.011	0.978	0.011	4	51	0.013	0.976	0.011	1
52	0.979	0.010	0.011	5	52	0.977	0.011	0.012	1
53	0.977	0.011	0.012	4	53	0.972	0.012	0.016	2
54	0.975	0.013	0.012	10	54	0.966	0.018	0.016	3
55	0.014	0.490	0.496	4	55	0.020	0.484	0.497	4
56	0.021	0.252	0.726	4	56	0.021	0.332	0.647	3
57	0.011	0.493	0.495	2	57	0.013	0.492	0.495	2
58	0.012	0.012	0.976	1	58	0.015	0.012	0.973	1
59	0.655	0.333	0.012	3	59	0.654	0.333	0.013	3
60	0.737	0.252	0.011	8	60	0.495	0.493	0.012	2
61	0.011	0.978	0.011	1	61	0.015	0.972	0.013	1
62	0.689	0.300	0.011	10	62	0.255	0.731	0.013	4
